# Supplementary material for: Endosomal maturation is controlled by the trimeric Bulli–Mon1–Ccz1 Rab7 GEF complex and the Rab5 GTPase-activating protein GAPsec
Source: J Cell Sci. 2026 May 11;139(9):jcs264460. doi: 10.1242/jcs.264460 (PMC13245906; doi:10.1242/jcs.264460)
Supplement: Supplementary information [file joces-139-264460-s1.pdf]

Supplementary Figure S1a CG1695, CG4041

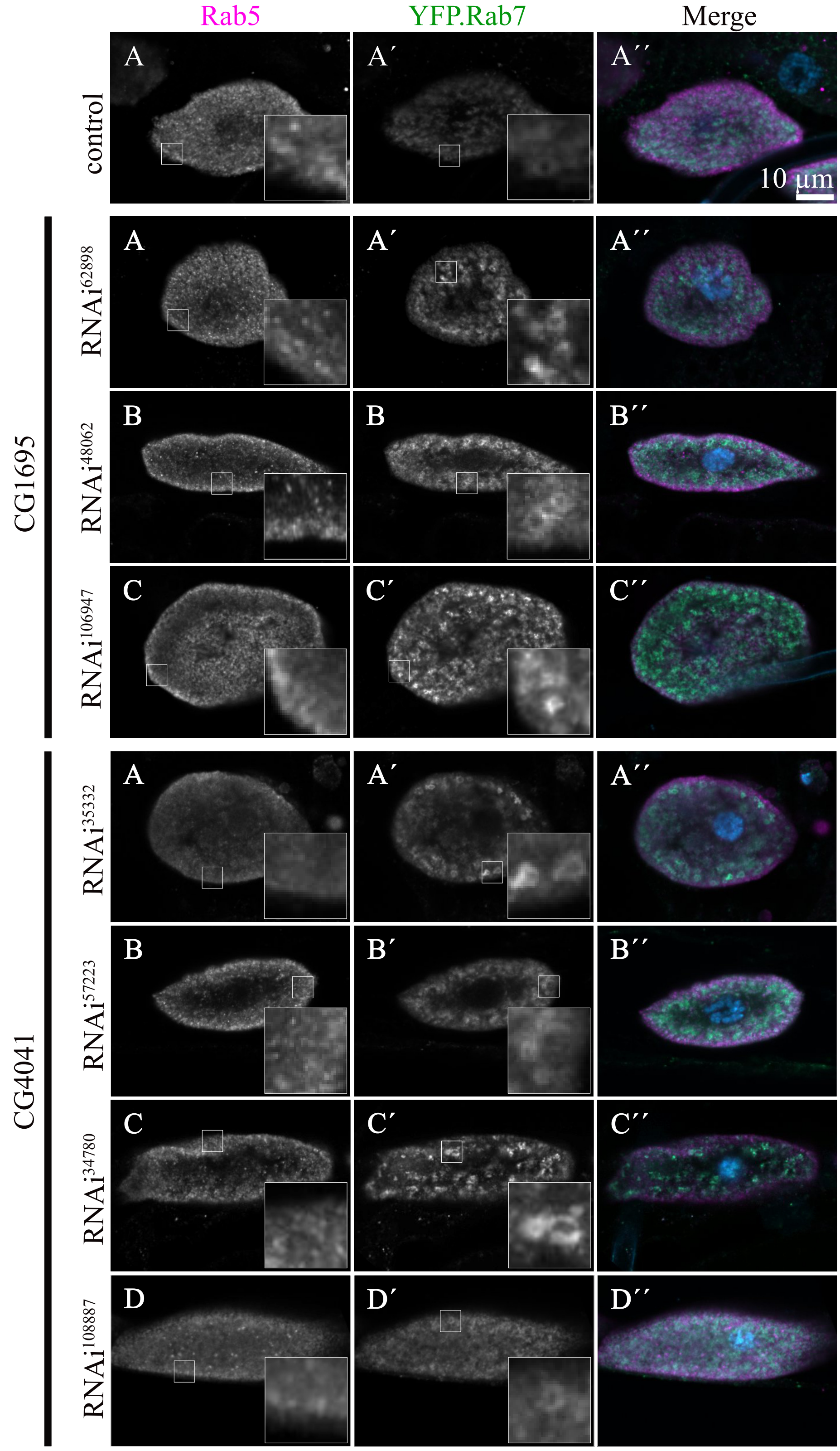

Supplementary Figure S1b CG4552 (TBC1D23), CG5337 (TBC1D16), CG5344 (Whacked)

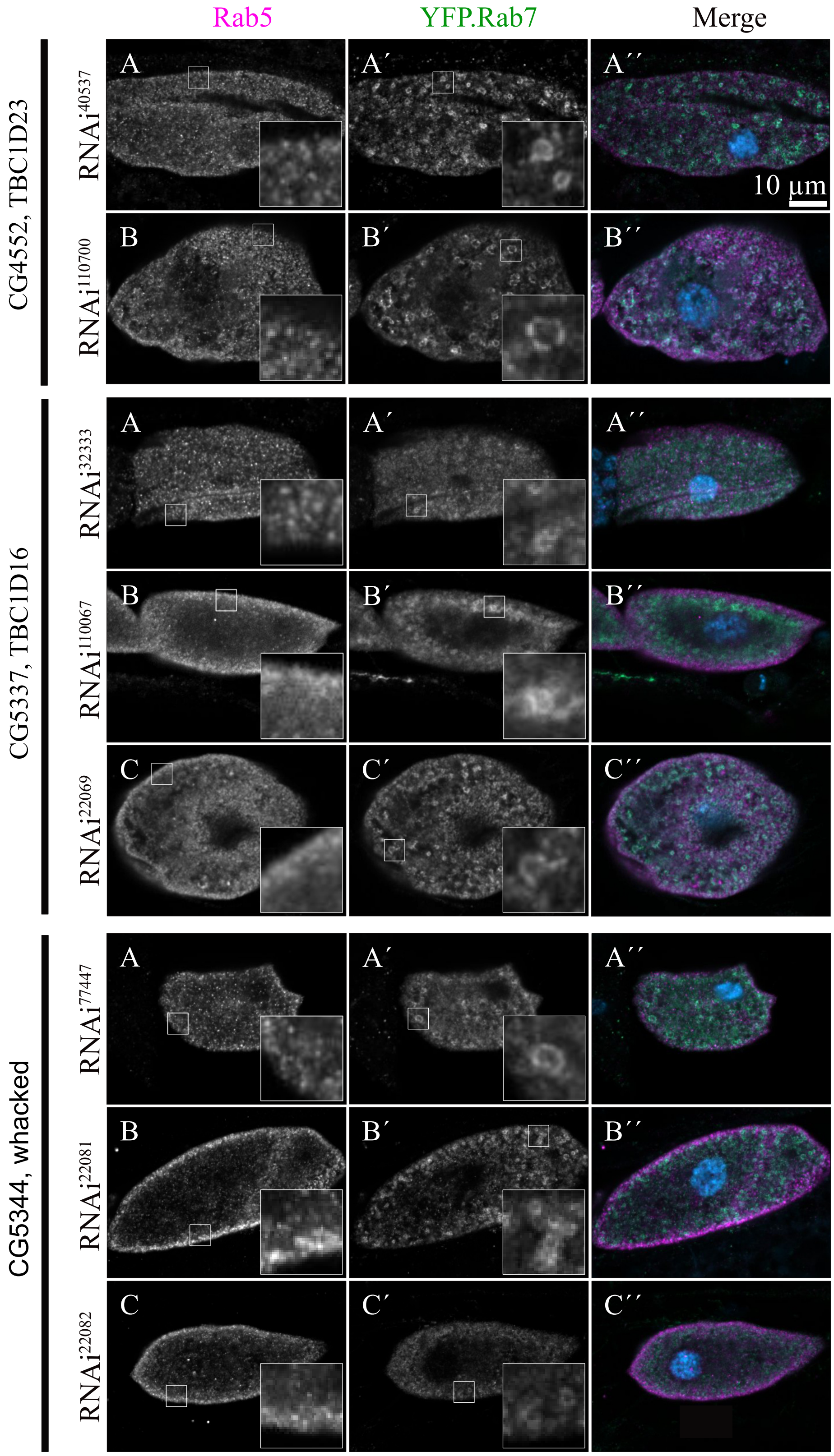

Supplementary Figure S1c CG5745 (TBC1D22), CG5916, CG5978 (GAPsec)

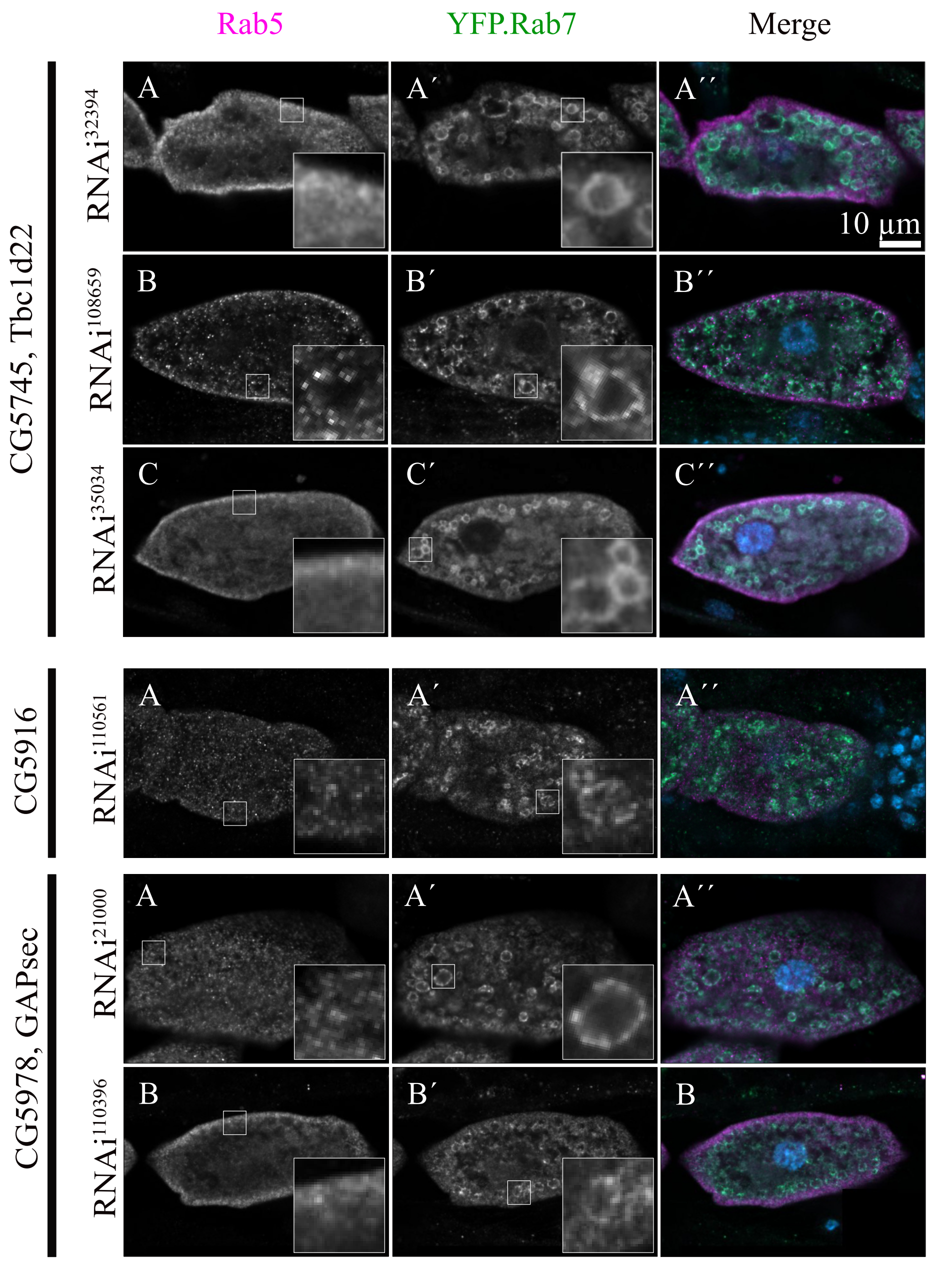

Journal of Cell Science • Supplementary information

Supplementary Figure S1d CG6182 (TBC1D7), CG7112 (GAPcenA), CG7324 (TBC1D8)

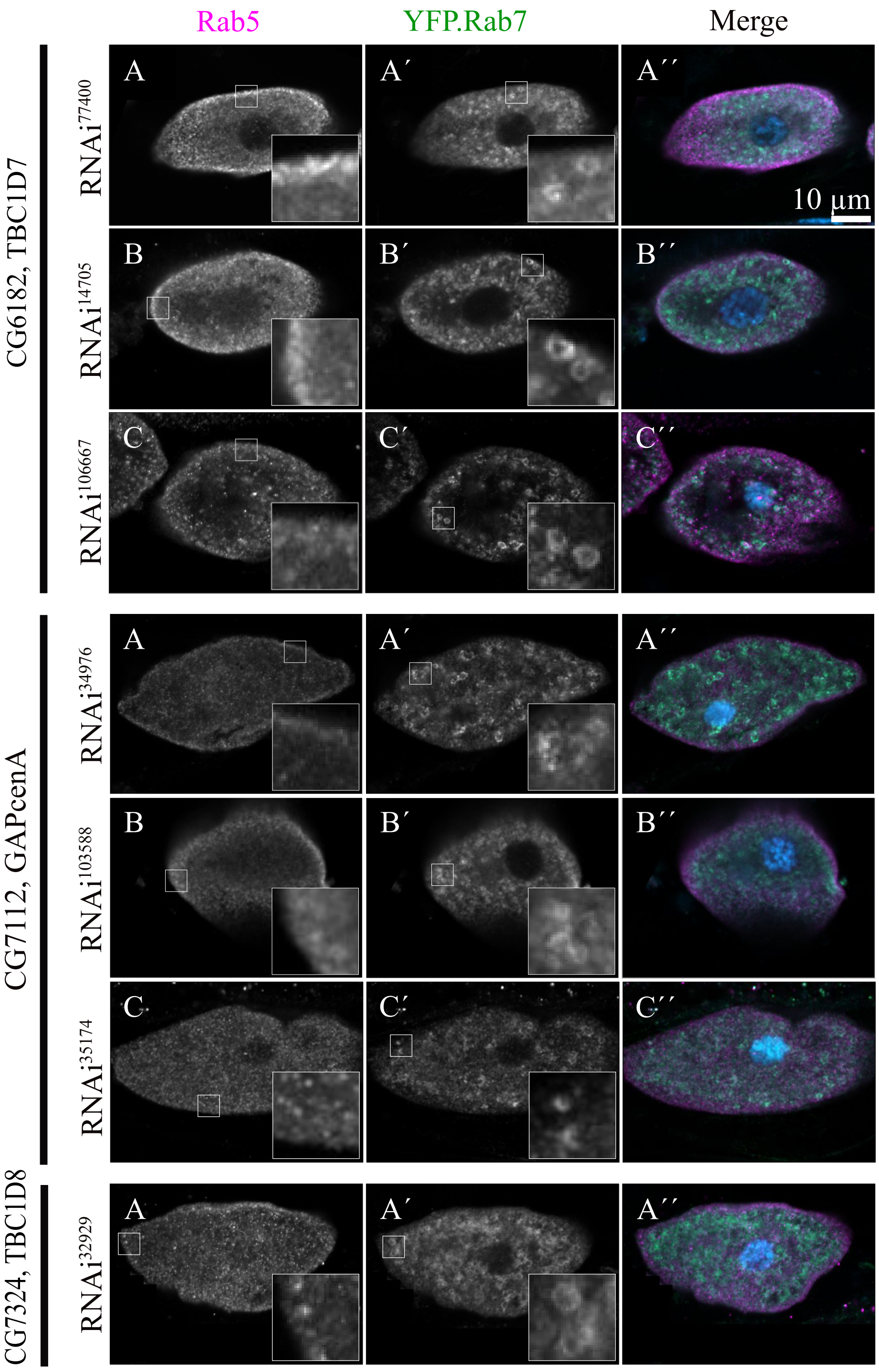

Journal of Cell Science • Supplementary information

Supplementary Figure S1e CG7742, CG8095 (RN-tree)

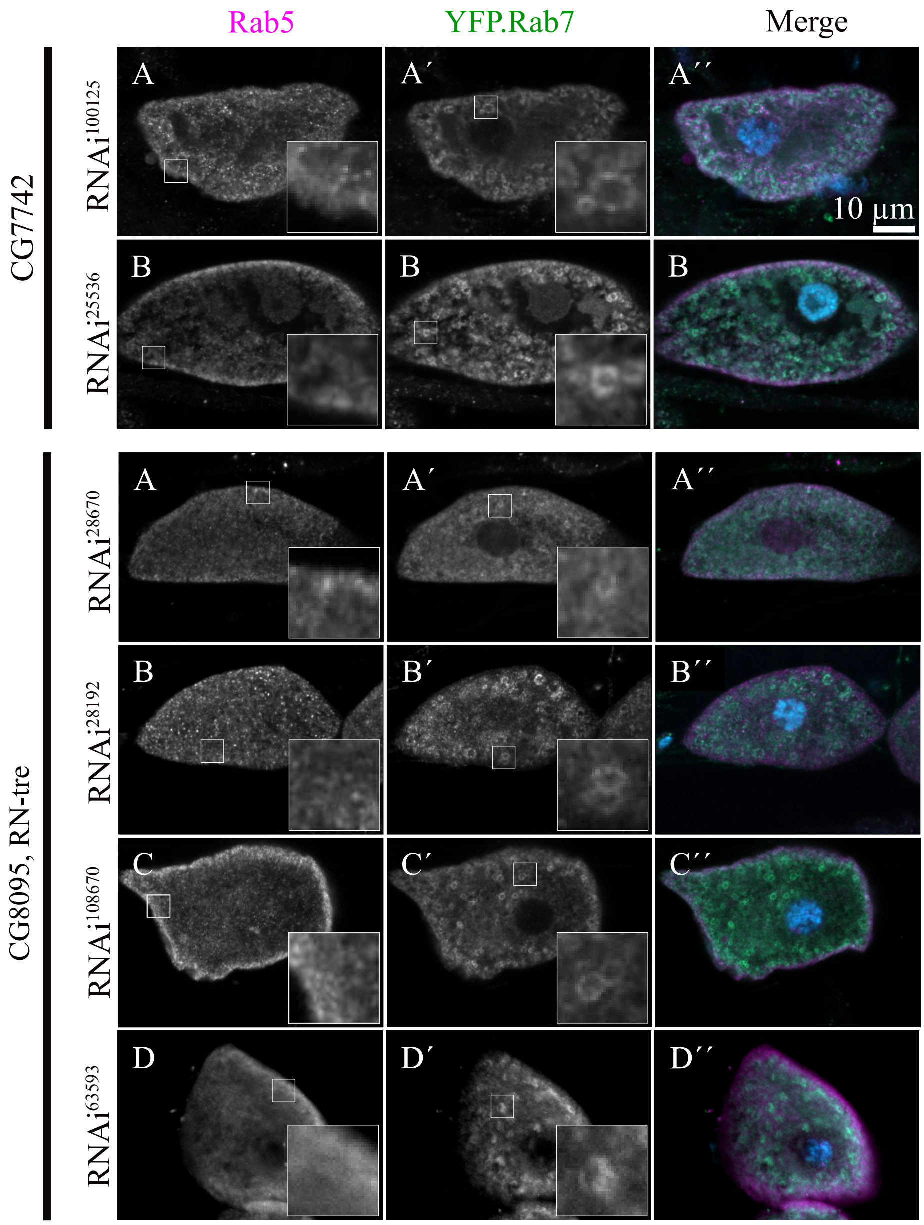

Supplementary Figure S1f CG8155, CG8449 (TBC1D5), CG9339 (skywalker)

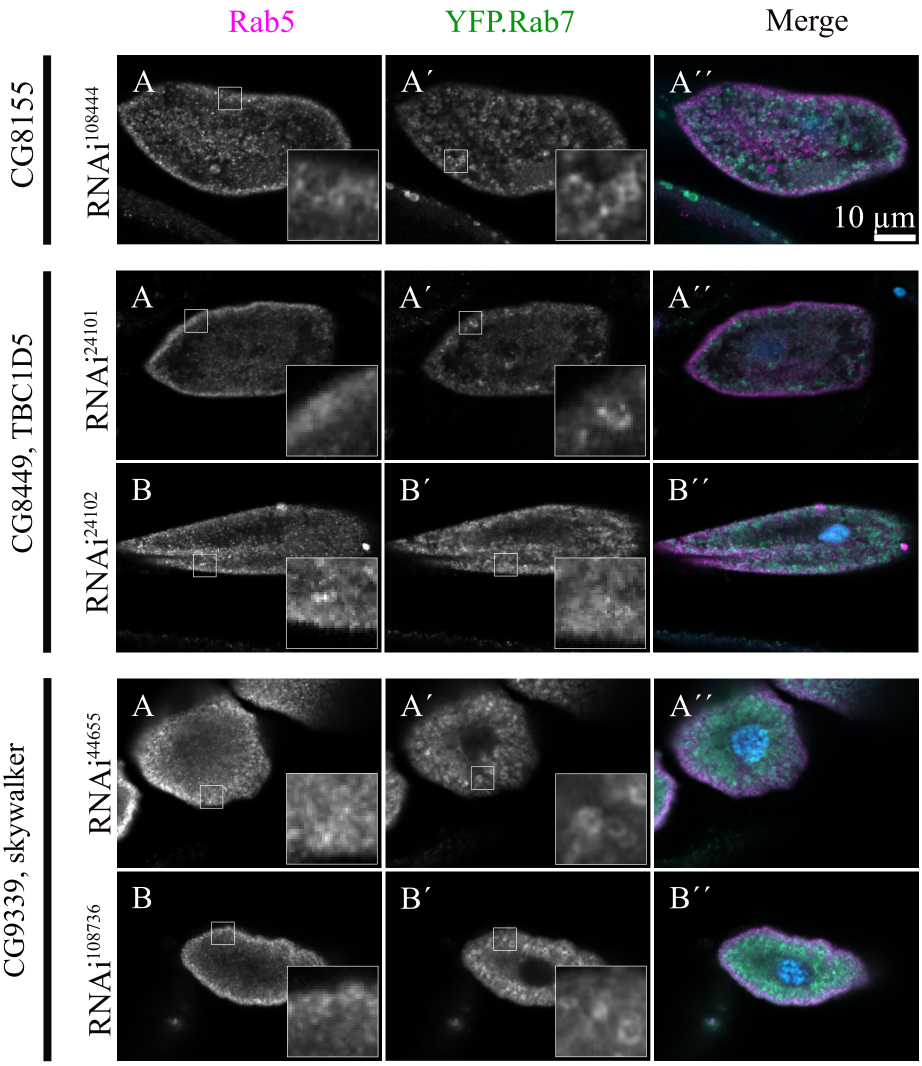

Journal of Cell Science • Supplementary information

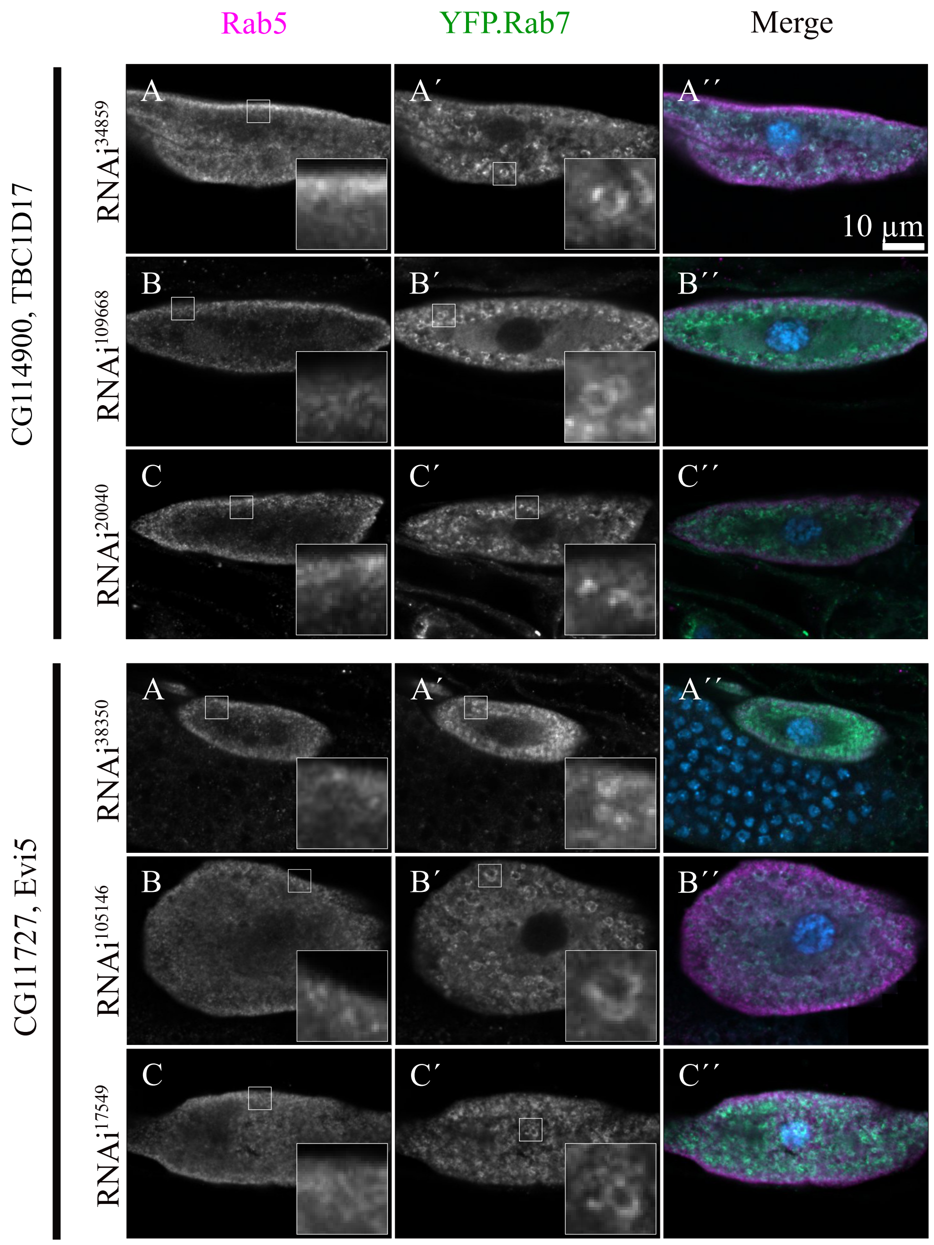

Supplementary Figure S1g CG9339 (skywalker), CG114900 (TBC1D17), CG11727 (Evi5)

Supplementary Figure S1h CG12241, CG16896 (TBC1D31), CG31935 (Rab3GAP1), CG32506 (trabuco)

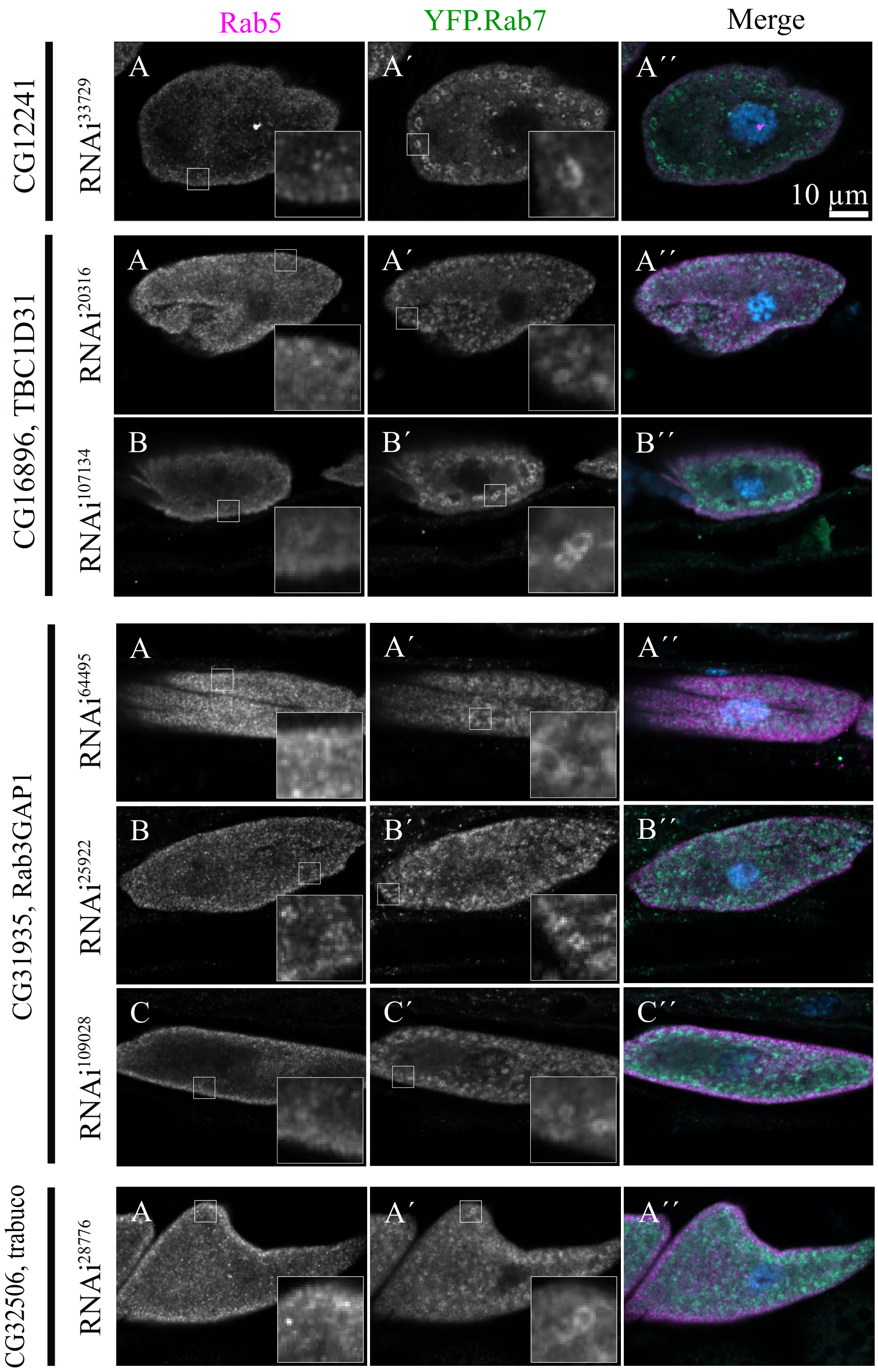

Supplementary Figure S1i   CG42612 (pollux), CG42795 (blobby)

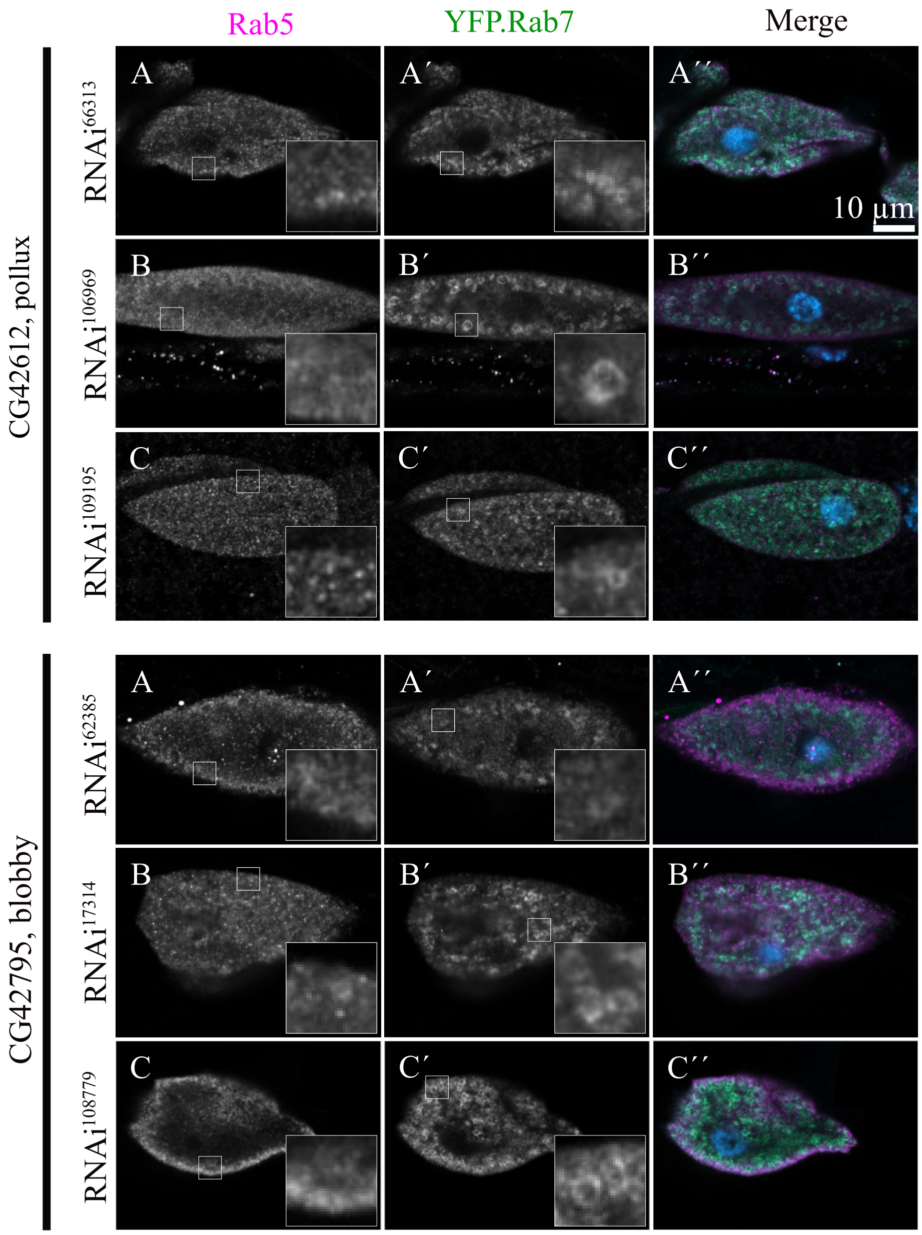

Fig. S1. Pericardial nephrocytes from dissected 3rd instar larvae of control flies and of flies, in which one of the potential *Drosophila* GAP genes was down-regulated by available RNAi-constructs, were stained for Rab5 (anti-Rab5) and Rab7 (anti-YFP.Rab7) to identify potential endosomal maturation defects.

Supplementary Figure 2a

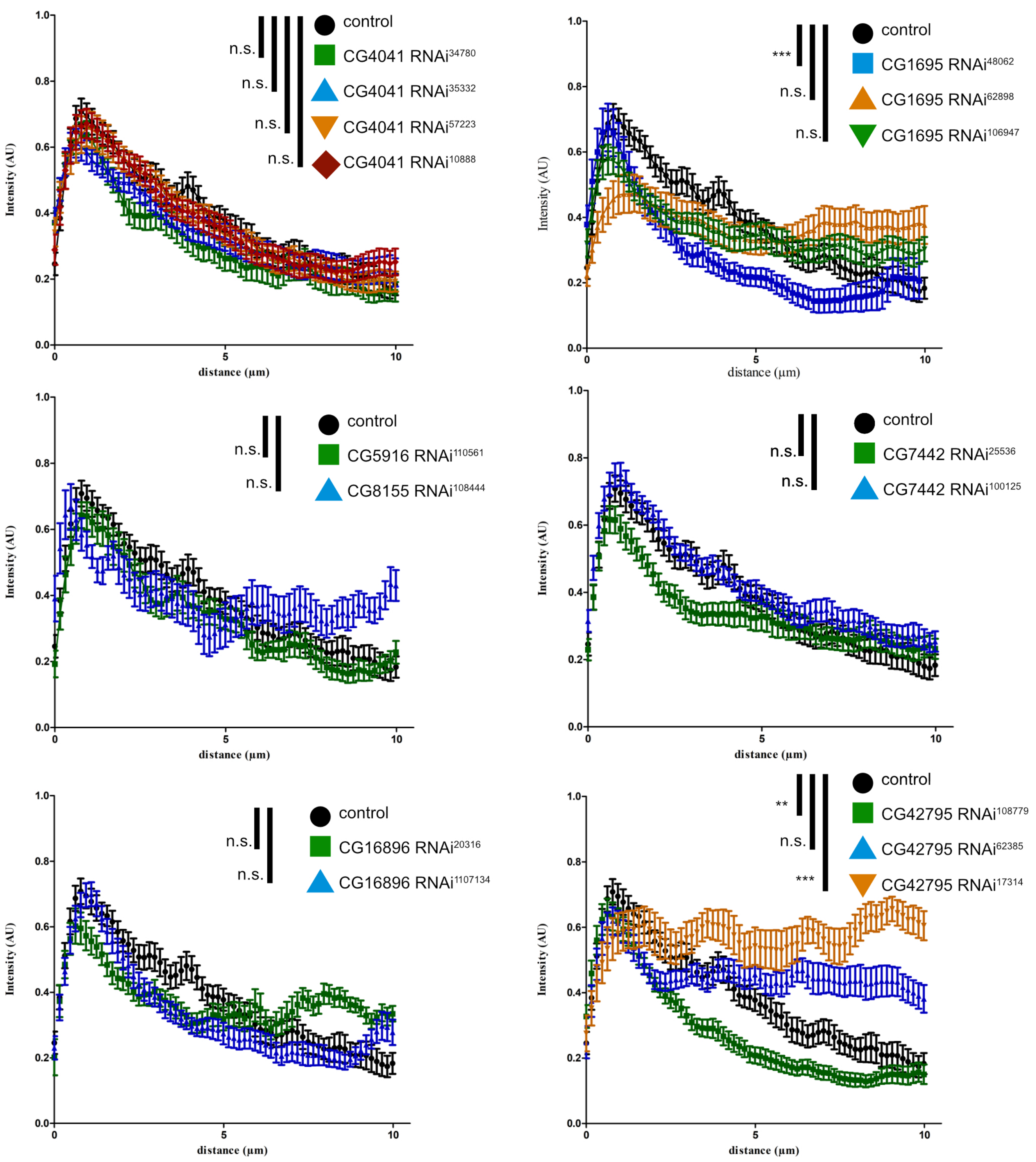

Journal of Cell Science • Supplementary information

Supplementary Figure 2b

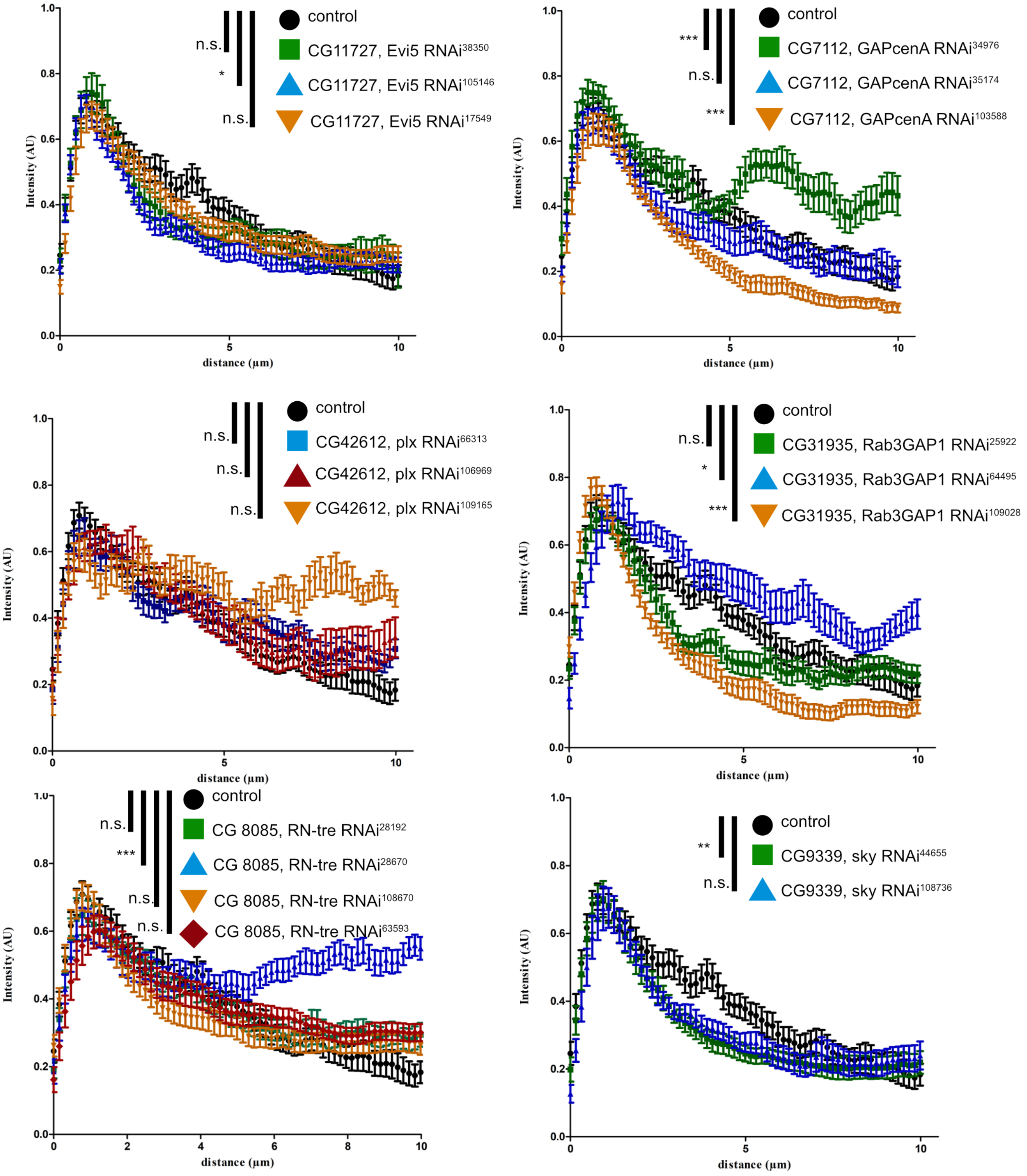

Journal of Cell Science • Supplementary information

Supplementary Figure 2c

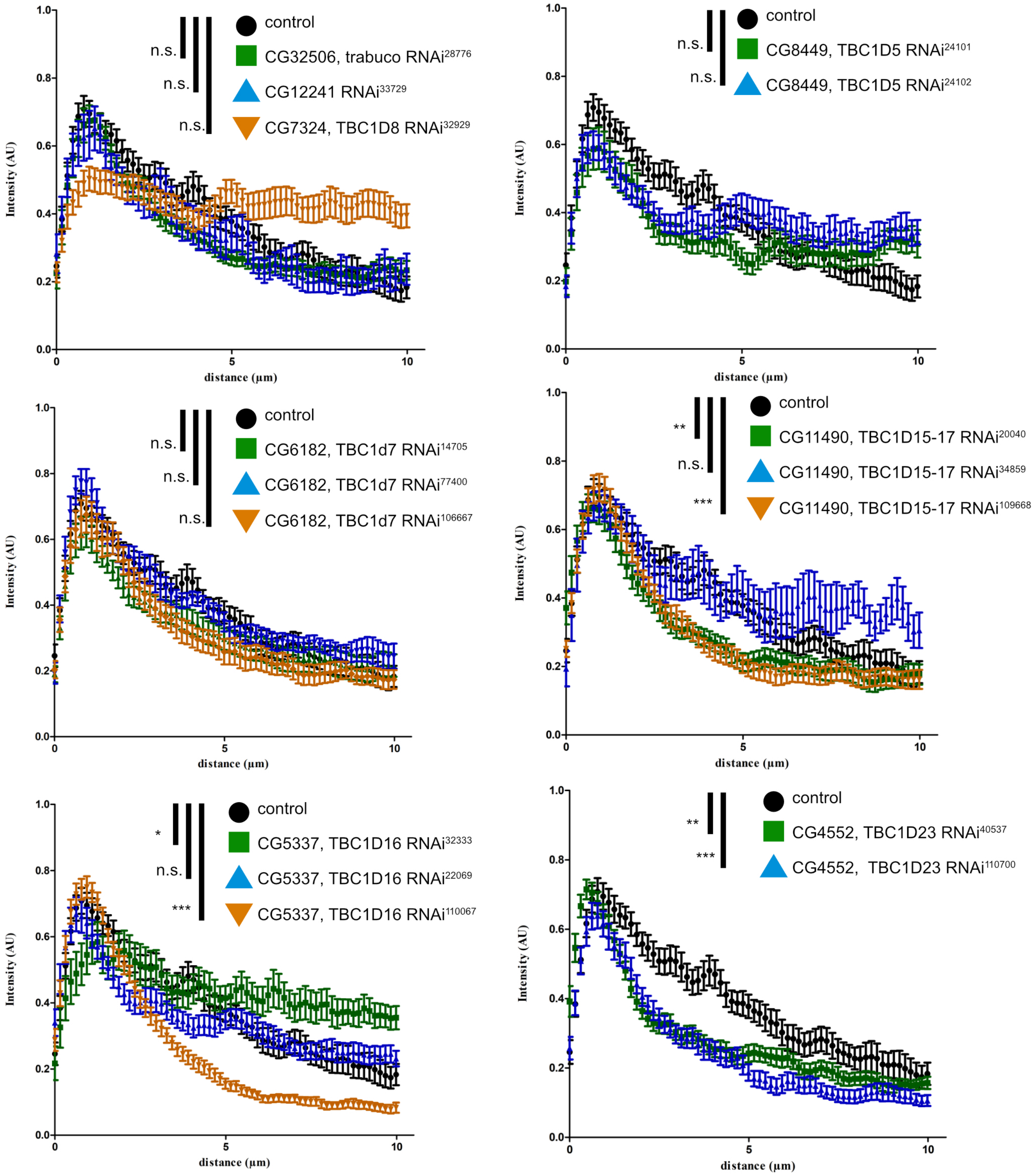

Journal of Cell Science • Supplementary information

Supplementary Figure 2d

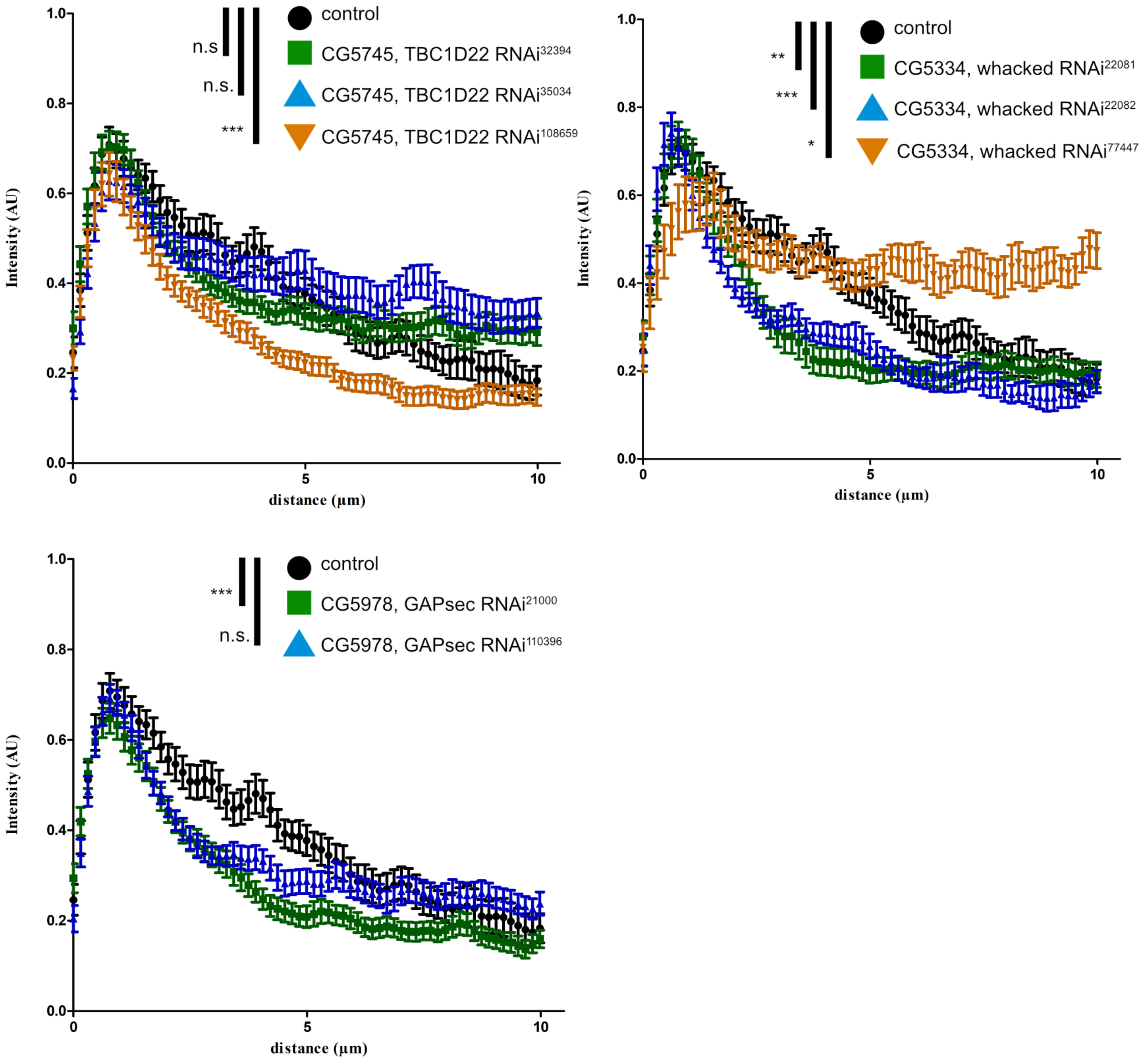

Journal of Cell Science • Supplementary information

Figure 2e

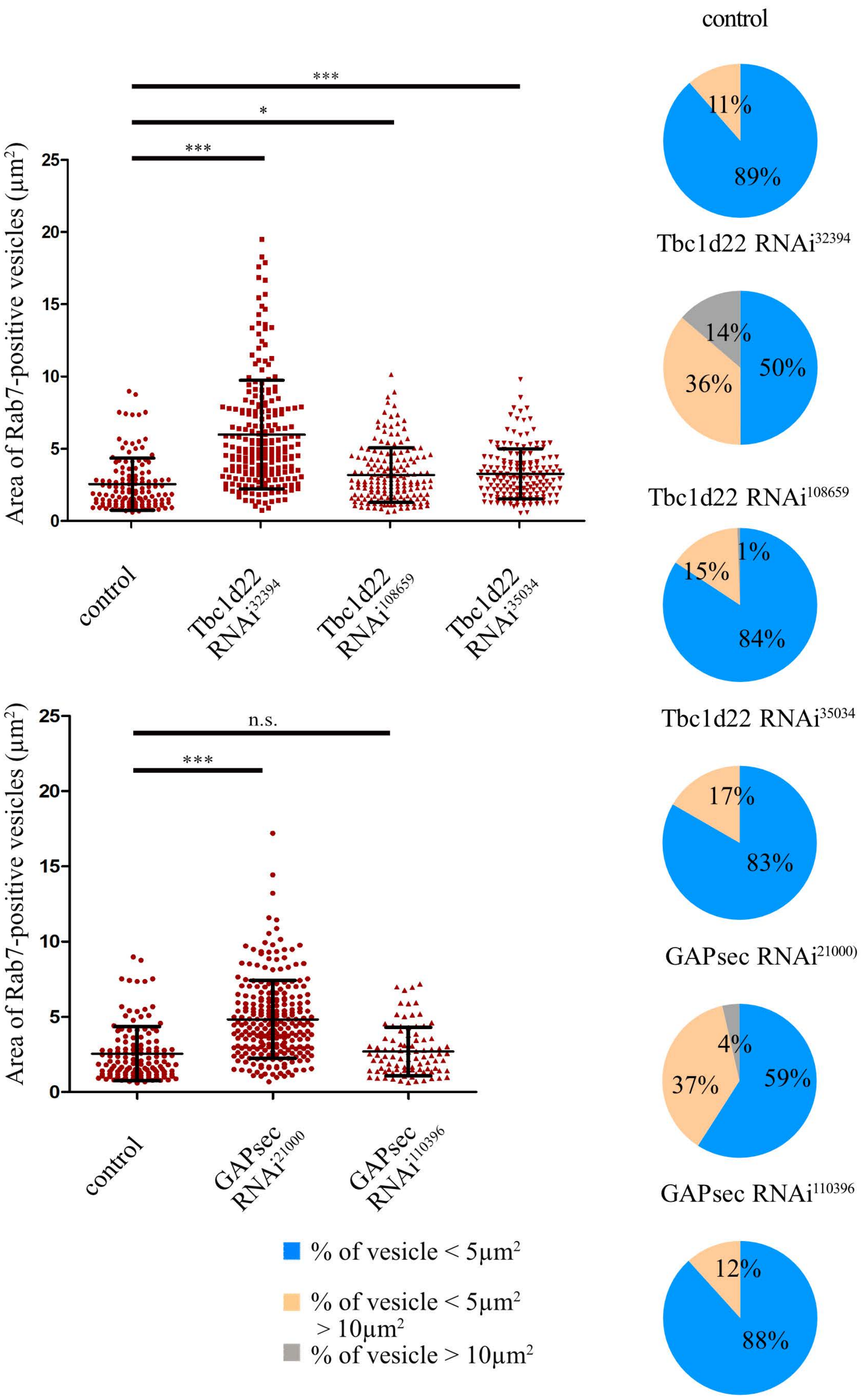
***
*
***
Area of Rab7-positive vesicles (μm²)
25
20
15
10
5
0
control
Tbc1d22 RNAi32394
Tbc1d22 RNAi108659
Tbc1d22 RNAi35034
n.s.
***
Area of Rab7-positive vesicles (μm²)
25
20
15
10
5
0
control
GAPsec RNAi21000
GAPsec RNAi110396
% of vesicle < 5μm²
% of vesicle < 5μm² > 10μm²
% of vesicle > 10μm²
control
11%
89%
Tbc1d22 RNAi32394
14%
50%
36%
Tbc1d22 RNAi108659
1%
15%
84%
Tbc1d22 RNAi35034
17%
83%
GAPsec RNAi21000)
4%
37%
59%
GAPsec RNAi110396
12%
88%

Fig. S2. Rab5 distribution in nephrocytes along a defined sector of 20 pixels in width and 10 µm in length. The starting point of teaching measurement was the outermost cell periphery. The control, *white*^{1118}, was always plotted in black. For example, if the localisation of the Rab5 vesicles in an RNAi line differs from the wild-type (control) distribution pattern in the cortex of a nephrocyte, the intensity curves differ. The "Polygon Selection" tool in ImageJ was used to manually encircle the Rab7-positive vesicles for the area analysis. All measured vesicles were inserted into the ROI Manager, and the area of each vesicle was calculated. Further data processing was performed using Microsoft Excel, and vesicles were subdivided into three groups according to their area: smaller than 5 µm^2, between 5 µm^2 and 10 µm^2 and larger than 10 µm^2. Prism was used for statistical analysis. The normal distribution was determined using the KS normality test, the D'Agestino & Pearson omnibus normality test, and the Shapiro-Wilk normality test. The total values of the different genotypes were then compared using the Kruskal-Wallis test. Asterisks indicate significance values. * $p<0.5$, n.s. not significant.
