# Supplementary material for: Endosomal maturation is controlled by the trimeric Bulli–Mon1–Ccz1 Rab7 GEF complex and the Rab5 GTPase-activating protein GAPsec
Source: J Cell Sci. 2026 May 11;139(9):jcs264460. doi: 10.1242/jcs.264460 (PMC13245906; doi:10.1242/jcs.264460)
Supplement: Supplementary file 1 [file joces-139-264460_review_history.pdf]

Endosomal maturation is controlled by the trimeric Bulli-Mon1-Ccz1 Rab7 GEF complex and the Rab5-GTPase activating protein GAPsec

Maren Janz, Maik Drechsler, Heiko Meyer, Vikeraman Sriram, Kim Michelle Simes, Elisa Frommhold, Nadia Füllbrunn, Lars Langemeyer, Christian Ungermann, Daniel Kümmel and Achim Paululat
DOI: 10.1242/jcs.264460

Editor: Robert Parton

Review timeline

Original submission:	17 September 2025
Editorial decision:	22 October 2025
First revision received:	25 February 2026
Editorial decision:	11 March 2026
Second revision received:	12 March 2026
Accepted:	13 March 2026

Original submission

First decision letter

MS ID#: jcs.264460

MS TITLE: Endosomal maturation is controlled by the trimeric Bulli-Mon1-Ccz1 GEF7 complex and Rab5-GTPase activating protein GAPsec

AUTHORS: Maren Janz; Maik Drechsler; Heiko Meyer; Vikeraman Sriram; Kim Michelle Simes; Elisa Frommhold; Nadia Füllbrunn; Lars Langemeyer; Christian Ungermann; Daniel Kümmel; Achim Paululat
ARTICLE TYPE: Research Article

Dear Dr Paululat,

We have now reached a decision on the above manuscript.

To see the reviewers' reports and a copy of this decision letter, please go to:

As you will see, the reviewers raise a number of substantial criticisms that prevent me from accepting the paper at this stage. They suggest, however, that a revised version might prove acceptable, if you can address their concerns. If you think that you can deal satisfactorily with the criticisms on revision, I would be pleased to see a revised manuscript. We would then return it to the reviewers.

Reviewer 1

SUMMARY OF THE ADVANCE MADE IN THIS PAPER AND ITS POTENTIAL SIGNIFICANCE TO THE FIELD

This manuscript by Janz et al. investigates the mechanisms controlling endosomal maturation in Drosophila nephrocytes, focusing on the trimeric Bulli-Mon1-Ccz1 (BuMC1) complex and a newly identified Rab5 GAP, GAPsec. The study builds upon the group's previous work describing BuMC1 as

© 2026. Published by The Company of Biologists under the terms of the Creative Commons Attribution License (https://creativecommons.org/licenses/by/4.0/).

a Rab7 GEF and provides new insight into how this complex coordinates with GAPsec to promote Rab5 inactivation and Rab7 activation during the endosome-to-lysosome transition.
The topic is timely and well aligned with the scope of Journal of Cell Science, which frequently features work on endosomal and lysosomal trafficking in Drosophila and mammalian systems. The manuscript is generally well written and the experimental strategy logical. However, some experimental gaps and missing references limit the strength of the conclusions. Addressing these issues will be essential before publication.

SUGGESTIONS TO AUTHORS

Major comments

1. The authors already possess a Bulli-GFP transgenic line, previously shown to localize to Rab7-positive endosomes (Dehnen et al., 2020). It is surprising that this reporter was not tested in Mon1, Ccz1, Rab7, Rab5, or GAPsec-depleted backgrounds. These analyses would directly demonstrate how Bulli recruitment depends on Rab5/Rab7 activity and whether GAPsec affects BuMC1 localization. Including such data would significantly strengthen the mechanistic conclusions.
2. The inclusion of TEM images is valuable, but the control nephrocyte images are of noticeably poorer quality and out of focus compared with mutant samples. Comparable image quality is required for proper interpretation. The authors should also present TEM data for GAPsec RNAi nephrocytes to evaluate whether this knockdown results in similar morphological defects.
3. TEM analysis reveals misplaced slit diaphragms in the examined mutants. However, these findings should be corroborated by confocal immunostaining of slit diaphragm markers (e.g., Pyd, Sns, Kirre, or Nephrin orthologs) and quantitative analysis. This would allow more objective evaluation of the reported morphological abnormalities.
4. The conclusion that GAPsec acts on Rab5 is intriguing but not yet firmly established. If GAPsec knockdown indeed acts via Rab5 inactivation, co-expression of constitutively active Rab5 (Rab5^CA-YFP) potentially could rescue, or at least reduce, the observed phenotypes. Testing this would strengthen the argument for GAPsec's role.
5. Several key Drosophila studies on CORVET/HOPS function and endosomal regulation are omitted. The following should be cited and discussed. PMIDs 27253064 and 31194677, which established the Drosophila miniCORVET complex and its Rab5 dependence. The ultrastructural similarities between Rab7 mutants (as shown in Fig. 7—figure supplement 1 of PMID 31194677) and Mon1/Ccz1 mutants reported here strongly support the present findings and should be acknowledged.
Besides Rab7 another small GTPases are also required for endosome maturation: Rab2 and Arl8 have both been implicated in nephrocytes (PMIDs 28483915, 29940804, 30590083). These should be referenced to place the current results into the broader context of small GTPase regulation.

Reviewer 2

In this manuscript Janz and colleagues aim to further dissect the function of a Bulli/Mon1/Ccz1 complex in endosomal maturation. The Mon/Ccz complex is a complex known to act as a GDP-GTP exchange factor for Rab7. In a previous publication, the lab had identified Bulli as a third subunit which is required for the complex GEF function in Drosophila. Building on their previous work, the authors report that mutations in all three subunits of the complex produce a similar phenotype, characterized by enlarged Rab7-positive endosomes and intracellular Rab5 accumulations as observed by light microscopy and electron microscopy. Using EM they further show defects in endocytic tubular structured names labyrinthine channels.

To test whether the effect seen in the BuMC1 mutants is due to hyperactivation of Rab5 and failure to recruit Rab7, they express constitutively active mutants of both GTPases and show that a Rab5 constitutively active mutant and a Rab7 constitutively inactive mutants lead to enlarged endosomes. As the BuMC1 mutants and Rab mutants appear to similarly affect endosomes, the authors conclude that the BuMC1 may be involved in the deactivation of Rab5 and the membrane recruitment of Rab7. This brings the authors to hypothesize that the BuMC1 complex may be responsible for the recruitment of a yet unknown Rab5 GAP. They therefore perform a RNAi screen to identify GAPs that again would yield the same phenotype of the BuMC1 mutants. They identify two GAPs whose knock down leads to the formation of enlarged Rab7 endosomes and inhibit endocytosis. One of them, GAPsec shows GAP activity against Rab5 in vitro.

© 2026. Published by The Company of Biologists under the terms of the Creative Commons Attribution License (https://creativecommons.org/licenses/by/4.0/).

While the identification of additional regulatory layers within the Rab5-to-Rab7 cascade is certainly an exciting topic, I have some concerns that several of the authors' conclusions are not fully supported by the presented data. I therefore recommend that the authors revise and further refine their model before the manuscript can be considered for publication.

Here are my major comments:

1) One of the claims of the authors is that the cellular phenotypes caused by Bulli and Mon1 and Ccz mutants in nephrocytes are similar (Figure 1). This is the base for furher experiments in the paper. If I look at the images and compare WT cells with the mutants I find the Mon and Ccz phenotype very different from the Bull mutant. I indeed see enlarged Rab7 endosomes with Rab5-positive inclusions (I wonder how Rab5 could end up in an enlarged endosomes) in the Bulli mutant. But I clearly see Rab5 defining slighly less enlarged Rab7 endosomes in the mon1 and ccz1 mutant and no Rab5 inclusions. This to me hints at differential effects of the 3 mutants on endosomal Rabs localization.
2) Figure 2: The contrast in A is much poorer than in B-D. I also qualitatively do not observe any difference of the labyrinthine channels. Maybe the authors could go into a bit more details on what one should notice and add some quantifications.
3) in Figure 3B, the author again claim that a Rab5 active mutant yields the same phenoty as the bulli mutant. I observe Rab7 is wrapping around the Rab5-positive membrane rather than Rab5 structures in Rab7 endosomes.
4) Page 9 line 3 "Importantly, under these conditions, we observed the characteristic BuMC1 phenotypes, with enlarged endosomes, partially reduced or absent Rab7 decoration (red arrows in Figure 4), and internalisation and accumulation of Rab5". I see enlarged Rab5 endosomes but the authors cannot concluse that Rab5 is enclosed in enlarged endosoms as seen with the bulli mutant.
5) The authors write in the abstract and throughout the text "We show here that BuMC1 aids in the inactivation of Rab5, releasing it from EEs to allow for maturation" but evidence logically linking BuMC1 and a GAP inactivating Rab5 is missing as this would require to show that the complex interacts and is responsible for the recruitment of the identified Rab5 GAP GAPsec.
6) How do the authors explain the variations in phenotypic manifestation of the various siRNA?

First revision

Author response to reviewers' comments

Rebuttal Letter jcs.264460

We thank both reviewers for their helpful and constructive comments. We addressed most of the issues raised and now provide a substantially revised version of our manuscript.

Reviewer 1:

Major comments

1. The authors already possess a Bulli-GFP transgenic line, previously shown to localize to Rab7-positive endosomes (Dehnen et al., 2020). It is surprising that this reporter was not tested in Mon1, Ccz1, Rab7, Rab5, or GAPsec-depleted backgrounds. These analyses would directly demonstrate how Bulli recruitment depends on Rab5/Rab7 activity and whether GAPsec affects BuMC1 localisation. Including such data would significantly strengthen the mechanistic conclusions.

Author’s response: In fact, the UAS-Bulli::GFP line was extremely helpful for our precipitation assays, which we presented in our publication by Dehnen et al. (2020). Ultimately, it was this Bulli::GFP line that identified the interaction with Mon1-Ccz1 and led to the discovery of the trimeric Bulli-Mon1-Ccz1 complex in flies. However, Bulli::GFP represents an overexpression

© 2026. Published by The Company of Biologists under the terms of the Creative Commons Attribution License (https://creativecommons.org/licenses/by/4.0/).

construct that, like any overexpressed protein, may be prone to mislocalization. Unfortunately, our antibody, which we generated against the Bulli protein, does not work in tissue stainings. Therefore, we currently have no adequate means to exclude possible mislocalisation of Bulli::GFP in cells. Since the subcellular localisation of Bulli is of great importance for further studies, and was also addressed by this reviewer (and also by several colleagues when presenting data), we will generate in the future CRISPR-Cas9 mediated GFP knock-in flies that express Bulli::GFP at endogenous levels. We hope that such a line produces a functional Bulli fusion protein suitable for subcellular localization studies.

2. The inclusion of TEM images is valuable, but the control nephrocyte images are of noticeably poorer quality and out of focus compared with mutant samples. Comparable image quality is required for proper interpretation. The authors should also present TEM data for GAPsec RNAi nephrocytes to evaluate whether this knockdown results in similar morphological defects.

Author's response: As requested, we replaced the TEM control image with higher-quality images **(new Figure 2)**. We also analyzed nephrocytes from the GAPsec RNAi lines in the TEM, as suggested **(see new Figure 7)**.

3. TEM analysis reveals misplaced slit diaphragms in the examined mutants. However, these findings should be corroborated by confocal immunostaining of slit diaphragm markers (e.g., Pyd, Sns, Kirre, or Nephrin orthologs) and quantitative analysis. This would allow more objective evaluation of the reported morphological abnormalities.

Author's response: As suggested, we performed immunostainings to analyze the labyrinthine channels. The slit diaphragms themselves appear normal in the TEM. We used Pyd and Spectrin as markers, which work very well in our hands, and analyzed the resulting stainings on a Zeiss AiryScan microscope. The data are shown in the **new Figure 3** and are discussed in the text.

4. The conclusion that GAPsec acts on Rab5 is intriguing but not yet firmly established. If GAPsec knockdown indeed acts via Rab5 inactivation, co-expression of constitutively active Rab5 (Rab5^CA-YFP) potentially could rescue, or at least reduce, the observed phenotypes. Testing this would strengthen the argument for GAPsec's role.

Author's response: Our hypothesis includes that GAPsec activity results in Rab5 inactivation. Therefore, the GAPsec knockdown should lead to reduced GAP function and consequently to prolonged Rab5 activity. Given this already increased Rab5 activity, we consider it unlikely that the additional expression of constitutively active Rab5 (Rab5^CA-YFP) could rescue the observed phenotypes.

5. Several key *Drosophila* studies on CORVET/HOPS function and endosomal regulation are omitted. The following should be cited and discussed. PMIDs 27253064 and 31194677, which established the *Drosophila* miniCORVET complex and its Rab5 dependence. The ultrastructural similarities between Rab7 mutants (as shown in Fig. 7—figure supplement 1 of PMID 31194677) and Mon1/Ccz1 mutants reported here strongly support the present findings and should be acknowledged.
Besides Rab7 another small GTPases are also required for endosome maturation: Rab2 and Arl8 have both been implicated in nephrocytes (PMIDs 28483915, 29940804, (Lőrincz et al., 2019)). These should be referenced to place the current results into the broader context of small GTPase regulation.

Author's response: The mentioned publications were included into the manuscript.

Reviewer 2

While the identification of additional regulatory layers within the Rab5-to-Rab7 cascade is certainly an exciting topic, I have some concerns that several of the authors' conclusions are not fully

© 2026. Published by The Company of Biologists under the terms of the Creative Commons Attribution License (https://creativecommons.org/licenses/by/4.0/).

supported by the presented data. I therefore recommend that the authors revise and further refine their model before the manuscript can be considered for publication.

Author's response: Thank you for the helpful and constructive comments. We have revised the manuscript in accordance with your suggestions and now present a significantly improved version.

Here are my major comments:

1) One of the claims of the authors is that the cellular phenotypes caused by Bulli and Mon1 and Ccz mutants in nephrocytes are similar (Figure 1). This is the base for furher experiments in the paper. If I look at the images and compare WT cells with the mutants I find the Mon and Ccz phenotype very different from the Bull mutant. I indeed see enlarged Rab7 endosomes with Rab5-positive inclusions (I wonder how Rab5 could end up in an enlarged endosomes) in the Bulli mutant. But I clearly see Rab5 defining slighly less enlarged Rab7 endosomes in the mon1 and ccz1 mutant and no Rab5 inclusions. This to me hints at differential effects of the 3 mutants on endosomal Rabs localization.

Author's response: We agree that the phenotypes in Mon1, Ccz1, and Bulli mutants are not completely identical. We discuss some of the possible explanations now within the discussion section (see tracked version of the manuscript). An important point is the fact that the dimeric Mon1/Ccz1 complex remains an active Rab7-GEF, even in the absence of the third component Bulli (see our paper Dehnen *et al*. 2020). This can explain, to some extent, why the Bulli phenotype appears milder in some aspects than the Mon1/Ccz1 phenotype.

2) Figure 2: The contrast in A is much poorer than in B-D. I also qualitatively do not observe any difference of the labyrinthine channels. Maybe the authors could go into a bit more details on what one should notice and add some quantifications.

Author's response: Both aspects were also mentioned by Reviewer 1. We have revised Figure 2 accordingly and included a completely new Figure 3 that focuses on the labyrinth channels.

3) in Figure 3B, the author again claim that a Rab5 active mutant yields the same phenoty as the bulli mutant. I observe Rab7 is wrapping around the Rab5-positive membrane rather than Rab5 structures in Rab7 endosomes.

Author's response: We have amended the wording and adopted it according to the reviewer's suggestion.

4) Page 9 line 3 "Importantly, under these conditions, we observed the characteristic BuMC1 phenotypes, with enlarged endosomes, partially reduced or absent Rab7 decoration (red arrows in Figure 4), and internalisation and accumulation of Rab5". I see enlarged Rab5 endosomes but the authors cannot conclude that Rab5 is enclosed in enlarged endosomes as seen with the bulli mutant.

Author's response: The paragraph has been reworded in accordance with the reviewer´s comment.

5) The authors write in the abstract and throughout the text "We show here that BuMC1 aids in the inactivation of Rab5, releasing it from EEs to allow for maturation" but evidence logically linking BuMC1 and a GAP inactivating Rab5 is missing as this would require to show that the complex interacts and is responsible for the recruitment of the identified Rab5 GAP GAPsec.

Author's response: That is ultimately correct, and it is on our to-do list to examine the roles of BuMC1 and GAPsec in detail. We included the following sentence into the discussion section to acknowledge this point: "Whether BuMC1 or Rab7 are directly involved in the recruitment of GAPsec or other Rab5-GAPs to endosomal membranes needs to be tested in future studies."
By the way: We are currently in the process of renewing a funded collaborative research center (called SFB in Germany), and the experiment mentioned above is a central part of my proposal.

© 2026. Published by The Company of Biologists under the terms of the Creative Commons Attribution License (https://creativecommons.org/licenses/by/4.0/).

6) How do the authors explain the variations in phenotypic manifestation of the various siRNA?

Author's response: This is a rather fundamental question. siRNA knockdown does rarely generate a null situation; residual activity of variable extent is commonly observed. In this regard, an analysis of the knockdown efficiency of a publicly available RNAi resource (TRiP collection, Harvard Medical School) revealed that >90% of the established lines exhibited residual gene expression of 25% or more (Perkins *et al*. 2015; Heigwer *et al*., 2018). Therefore, the mechanisms by which a cell compensates for a loss of 70%, 80%, or 90% of GAP activity (or other endocytic factors) may vary and are difficult to predict. However, they can explain the observed variations in phenotypic manifestation.
We included this information in the results section.

Second decision letter

MS ID#: jcs.264460R1

MS TITLE: Endosomal maturation is controlled by the trimeric Bulli-Mon1-Ccz1 GEF7 complex and Rab5-GTPase activating protein GAPsec

AUTHORS: Maren Janz; Maik Drechsler; Heiko Meyer; Vikeraman Sriram; Kim Michelle Simes; Elisa Frommhold; Nadia Füllbrunn; Lars Langemeyer; Christian Ungermann; Daniel Kümmel; Achim Paululat
ARTICLE TYPE: Research Article

Dear Dr Paululat,

We have now reached a decision on the above manuscript.

As you will see, the reviewers gave favourable reports but one reviewer raised an important point that will require amendments to the text of your manuscript. Once those are completed to our satisfaction I anticipate being able to accept your manuscript promptly.

Reviewer 1

Thank you for thoroughly addressing my previous comments and providing clear, honest responses. The revisions have strengthened the manuscript, and I have no further concerns.

Reviewer 2

I am satisfied with the authors answers to my points 2-6.

However, the authors have failed to fully address my major concern 1. I initially raised the concern that the phenotypes observes in figure 1 for the various mutants are rather different. And I am perfectly ok with the explanation they provide in the discussion. Still no changes were made in the paragraphs describing Figure 1. The sentence "Similar to bulli6-61 mutants, we observed that the lack of either Mon1 or Ccz1 results in intravesicular Rab5 deposits throughout the cells (Figure 1B-D)" is misleading because as I pointed out before these phenotypes are rather different. I am not expecting any further experiments, only that the authors describe more precisely their observations.

Second revision

© 2026. Published by The Company of Biologists under the terms of the Creative Commons Attribution License (https://creativecommons.org/licenses/by/4.0/).

Author response to reviewers' comments

In this revision, only one sentence has been added, as requested by one reviewer. The sentence is marked in the highlighted version.

Third decision letter

MS ID#: jcs.264460R2

MS Title: Endosomal maturation is controlled by the trimeric Bulli-Mon1-Ccz1 GEF7 complex and Rab5-GTPase activating protein GAPsec

Authors: Maren Janz; Maik Drechsler; Heiko Meyer; Vikeraman Sriram; Kim Michelle Simes; Elisa Frommhold; Nadia Füllbrunn; Lars Langemeyer; Christian Ungermann; Daniel Kümmel; Achim Paululat
Article Type: Research Article

Dear Dr Paululat,

I am happy to tell you that your manuscript has been accepted for publication in Journal of Cell Science, pending standard publication integrity checks.

© 2026. Published by The Company of Biologists under the terms of the Creative Commons Attribution License (https://creativecommons.org/licenses/by/4.0/).
